# Characterization of Cancer Stem Cells in Colon Adenocarcinoma Metastasis to the Liver

**DOI:** 10.3389/fsurg.2017.00076

**Published:** 2018-01-22

**Authors:** Hugo N. Humphries, Susrutha K. Wickremesekera, Reginald W. Marsh, Helen D. Brasch, Shreeja Mehrotra, Swee T. Tan, Tinte Itinteang

**Affiliations:** ^1^Gillies McIndoe Research Institute, Wellington, New Zealand; ^2^Upper Gastrointestinal, Hepatobiliary and Pancreatic Section, Department of General Surgery, Wellington Regional Hospital, Wellington, New Zealand; ^3^University of Auckland, Auckland, New Zealand; ^4^Wellington Regional Plastic, Maxillofacial and Burns Unit, Hutt Hospital, Wellington, New Zealand

**Keywords:** cancer stem cells, colorectal, colon, cancer, adenocarcinoma, liver, metastasis

## Abstract

**Background:**

Fifty percent of colorectal cancer (CRC) patients develop liver metastasis. This study identified and characterized cancer stem cells (CSCs) within colon adenocarcinoma metastasis to the liver (CAML).

**Methods:**

3,3-Diaminobenzidine immunohistochemical (IHC) staining was performed on nine CAML samples for embryonic stem cell (ESC) markers OCT4, SOX2, NANOG, c-Myc, and KLF4. Immunofluorescence (IF) IHC staining was performed to investigate coexpression of two markers. NanoString mRNA expression analysis and colorimetric *in situ* hybridization (CISH) were performed on four snap-frozen CAML tissue samples for transcript expression of these ESC markers. Cells stained positively and negatively for each marker by IHC and CISH staining were counted and analyzed.

**Results:**

3,3-Diaminobenzidine IHC staining, and NanoString and CISH mRNA analyses demonstrated the expression of OCT4, SOX2, NANOG, c-Myc, and KLF4 within in all nine CAML samples, except for SOX2 which was below detectable levels on NanoString mRNA analysis. IF IHC staining showed the presence of a SOX2^+^/NANOG^+^/KLF4^+^/c-Myc^+^/OCT^−^ CSC subpopulation within the tumor nests, and a SOX2^+^/NANOG^+^/KLF4^+^/c-Myc^+^/OCT4^−^ CSC subpopulation and a SOX2^+^/NANOG^+^/KLF4^+^/c-Myc^+^/OCT4^+^ CSC subpopulation within the peritumoral stroma.

**Conclusion:**

The novel finding of three CSC subpopulations within CAML provides insights into the biology of CRC.

## Introduction

Colorectal cancer (CRC) is the third most common cancer and cause of cancer deaths in both men and women in the United States with 90% being adenocarcinoma (1). In 2015, CRC accounted for 9.7% of all cancer in the world with approximately 814,000 cases in men and 664,000 cases in women (2). In New Zealand, CRC was the second most common cancer, with 3,075 newly registered cases in 2013 (3).

Colorectal cancer has been attributed to a complex heterogeneous interaction of lifestyle factors and genetic predisposition. Regular consumption of red meats and alcohol, and inflammatory bowel disease such as ulcerative colitis, are known risk factors for CRC (4). Three major genetic/molecular pathways have been implicated in the development of CRC: chromosomal instability pathway (CIP), microsatellite instability pathway (MSI), and CpG island methylator phenotype pathway (CIMP) (2, 5, 6). Of these, CIP appears to be the most relevant in the pathogenesis of CRC (2, 5, 6).

The liver is a common metastatic site for CRC. Approximately 20% of CRC patients present with liver metastasis at the time of diagnosis (7) and overall 50% of CRC patients develop liver metastasis during the course of their disease, accounting for 2/3 of the disease-related deaths (7, 8).

The survival time for untreated CRC patients with liver metastasis remains at 5–20 months (8). Liver resection is the mainstay treatment for CRC metastatic to the liver (CRCML) and it is the only intervention that significantly increases life expectancy, with a 5-year survival rate of up to 58% (7, 8). However, only 20% of patients with CRCML are operable at time of diagnosis (7). Chemotherapy plays a palliative role if used as a primary treatment, so recent focus has been on neoadjuvant chemotherapy to downstage the metastatic liver disease to increase operability (7, 8).

Stem cells are essential to the growth and development as well as the maintenance of human tissues, with the key abilities of self-renewal and maintenance of pluripotency, and differentiation through asymmetrical division (9–11).

The cancer stem cell (CSC) concept of cancer proposes that a subpopulation of cells within the cancer possess properties of embryonic stem cells (ESCs) and are thought to be the quintessential drivers of carcinogenesis due to their ability to promote angiogenesis, invasion, metastasis, and resistance to apoptosis as well as hijacking stem cell properties (12, 13).

OCT4 ensures the survival of ESCs through maintenance of the pluripotent state and self-renewal capabilities (11, 13, 14). SOX2 and OCT4 act in partnership and indispensably together to regulate the expression of genes specific for maintaining stem cell pluripotency and self-renewal (11, 15). NANOG also contributes to the transcriptional factor regulatory network involved in maintaining the key properties of stem cells, facilitated through control of genes maintaining pluripotency (16). c-Myc is a proto-oncogene with a significant role in fundamental cellular process and is involved in generation of induced pluripotent cells through activation of downstream targets which enhance proliferation and transformation of cells (16, 17). KLF4 is involved in regulation of the cell cycle, somatic cell reprogramming, and pluripotent characteristics (18).

A number of other stem cell markers associated with CRC CSCs have also been reported in the literature. CD44 is a cell surface glycoprotein with a number of roles in regulating cellular structure and cell–cell interactions (19, 20). Overexpression of CD44 has been implicated in early colorectal carcinogenesis, with CD44 knockdown preventing this process (19, 20). Leucine-rich repeat-containing G protein-coupled receptor 5 (LGR5) is a marker specific to crypt base columnar cells (CBCs) which are undifferentiated stem cells that give rise to the colonic mucosa (19). LGR5 knockdown results in tumor regression (21). Epithelial cell adhesion molecule (EpCAM) is a marker unique to epithelial cells and consequently tumors of epithelial morphology, with functions in cell signaling, and regulating processes such as proliferation, differentiation and metastasis (19). EpCAM^+^ cells have also been extensively characterized as CRC CSCs (19).

This study aimed to identify and characterize CSCs within colon adenocarcinoma metastasis to the liver (CAML) using ESC markers OCT4, SOX2, NANOG, c-Myc, and KLF4 by immunohistochemical (IHC) staining, colorimetric *in situ* hybridization (CISH), and NanoString mRNA expression analysis.

## Materials and Methods

### Tissue Samples

Colorectal adenocarcinoma metastasis to the liver samples from nine male patients aged 50–80 (mean, 65) years from the Gillies McIndoe Research Institute Tissue Bank were used for this study which was approved by the Central Health and Disabilities Ethics Committee (ref. no. 15/CEN/106). Written informed consent was obtained from patients included in this study.

### Histochemical and IHC Staining

Hematoxylin and eosin (H&E) staining was performed on 4μm-thick formalin-fixed paraffin-embedded sections of nine samples of CAML to confirm the presence of the tumor on the slides by an anatomical pathologist (HDB). 3,3-Diaminobenzidine (DAB) IHC staining was then performed on these sections for CD44 (1:1,500; cat# MRQ-13, Cell Marque, Rocklin, CA, USA), OCT4 (1:30; cat# MRQ-10, Cell Marque), SOX2 (1:200; cat# PA1-094, Thermo Fisher Scientific, Rockford, IL, USA), KLF4 (1:200; cat# NBP2-24749SS, Novus Biologicals LLC, Littleton, CO, USA), NANOG (1:100; cat# ab80892, Abcam, Cambridge, MA, USA), and c-Myc (1:1,000; ca# 9E10, Abcam) as previously described (22). All DAB IHC-stained slides were mounted in Surgipath Micromount (Leica, Nussloch, Germany). To confirm coexpression of two proteins, two representative samples of CRCML from the original cohort of nine samples used for DAB IHC staining underwent immunofluorescence (IF) IHC staining. Vectafluor Excel antimouse 488 (ready-to-use; cat#VEDK2488, Vector Laboratories, Burlingame, CA, USA) and Alexa Fluor antirabbit 594 (1:500; cat#A21207, Life Technologies, Carlsbad, CA, USA) were used to detect the combinations. All IF IHC-stained slides were mounted in Vecta Shield Hardset mounting medium with 4′,6′-diamino-2-phenylindone (Vector Laboratories). All antibodies were diluted in Bond primary diluent (Leica). All DAB and IF IHC staining was performed using the Leica Bond Rx auto-stainer (Leica), as previously described (22).

Positive human control tissues used for the primary antibodies were seminoma for OCT4 and NANOG (23), skin for SOX2 (24), breast cancer for KLF4 (25) and prostate for c-Myc (26). A negative CAML control sample was prepared for DAB IHC staining using an IgG isotype control (ready-to-use; cat#IR600, Dako, Santa Clara, CA, USA). Negative controls for IF IHC staining was performed using a section of glioblastoma tissue with the combined use of primary isotype mouse (ready-to-use; cat# IR750, Dako, Copenhagen, Denmark) and rabbit (read-to-use; cat# IR600, Dako) antibodies.

### Image Analysis

3,3-Diaminobenzidine IHC-stained slides were viewed and images were captured using the Olympus BX53 microscope fitted with an Olympus DP21 digital camera (Olympus, Tokyo, Japan). IF IHC-stained slides were viewed and imaged using the Olympus FV1200 biological confocal laser-scanning microscope and processed with cellSens Dimension 1.11 software using 2D deconvolution algorithm (Olympus).

### NanoString mRNA Expression Analysis

RNA was extracted from six snap-frozen samples of CAML from the same cohort of nine patients used for DAB IHC staining, was analyzed using NanoString nCounter™ Gene Expression Assay (NanoString Technologies, Seattle, WA, USA). Total RNA was extracted using the MagJET RNA kit (Thermo Fisher Scientific) with the protocol adapted for tissue, and run on a KingFisher Duo machine (Thermo Fisher Scientific). The RNA samples were then quantitated on a Qubit^®^ 2.0 fluorometer (Invitrogen, Life Technologies) and were subject to RNA integrity analysis *via* the 2100 Bioanalyzer Instrument (Agilent Technologies). The samples then underwent NanoString nCounter gene expression assay performed by New Zealand Genomics (Dunedin, New Zealand) according to the manufacturer’s protocol. Probes for the genes encoding OCT4 (POU5F1, NM_002701.4), SOX2 (NM_003106.2), NANOG (NM_024865.2), KLF4 (NM_004235.4), c-Myc (NM_002467.3), and the housekeeping gene GUSB (NM_000181.1) were designed and synthesized by NanoString Technologies. Raw data were analyzed using nSolver™ software (NanoString Technologies) using standard settings and normalized against the housekeeping gene.

### Colormetric *In Situ* Hybridization

The 4μm-thick formalin-fixed paraffin-embedded CAML sections of six patients from the original cohort of nine patients used for DAB IHC staining were used for CISH staining using the Leica Bond Rx autostainer and detected using the ViewRNA eZ Detection kit (Affymetrix, CA, USA) as previously described (27). Probes for the stem cell markers c-Myc (NM_002467), KLF4 (NM_004235), NANOG (NM_024865), SOX2 (NM_003106), and OCT4 (POU5F1, NM_002701) were obtained from Affymetrix. Positive controls were demonstrated on human tissues: breast cancer for KLF4, normal prostate for c-Myc, skin for SOX2, and seminoma for OCT4 and NANOG. Negative control was demonstrated on a sample of *Bacillus* (NM_L38424).

### Cell Counting

Counting of cells with nucleus or cytoplasm that stained positively for OCT4, SOX2, NANOG, KLF4, and c-Myc was each performed in six fields of view at 400× magnification representative of both tumor nests (TNs) and the peritumoral stroma for each of the original nine samples of CAML used for DAB IHC staining. In each field of view, cells within the TNs and those within the peritumoral stroma were counted separately and hence images were taken to incorporate areas of tissue containing cells both within the TNs and the peritumoral stroma.

Cell counting for CISH-stained slides was performed at 1,000× magnification which meant that cells within the TNs and those within the peritumoral stroma could not be incorporated in a single image and hence images were taken separately for cells within the TNs and those within the peritumoral stroma.

### Statistical Analyses

Statistical analysis for comparison of markers demonstrated by DAB IHC and CISH staining was performed to determine significance. Total counts of cells that stained positively and those that stained negatively for each ESC marker within the TNs and the peritumoral stroma were collated. The proportion of positive staining cells was calculated for each marker in both the TNs and peritumoral stroma. χ^2^ tests were then carried out using the Quantpsy (www.quantpsy.org) software to determine statistical significance with regards to the proportional expression of each marker, and related *t*-tests were used to compare the proportional expression of each ESC marker within the TNs against those within the peritumoral stroma using SPSS V.24.

## Results

### Histochemical and DAB IHC Staining

Hematoxylin and eosin staining confirmed the presence of CAML on the slides, distinguishing CAML from normal liver architecture and identifying two distinct populations of cells within adenocarcinoma glandular TNs and the peritumoral stroma (Figure S1A in Supplementary Material).

Membranous staining of CD44 was demonstrated on the cells within the TNs (Figure S1B in Supplementary Material, brown). Cytoplasmic staining for OCT4 (Figure S1C in Supplementary Material, brown) was observed on cells within the peritumoral stroma but not those within the TNs, with the cells that stained positively appearing in distinct clusters throughout the tissue. NANOG (Figure S1D in Supplementary Material, purple), SOX2 (Figure S1E in Supplementary Material, brown), and c-Myc (Figure S1F in Supplementary Material, brown) were expressed on the cells within the TNs (Figures S1D–F in Supplementary Material, *arrows*) and peritumoral stroma (Figures S1D–F in Supplementary Material, *arrowheads*). Cytoplasmic staining of KLF4 (Figure S1G in Supplementary Material, purple, *arrows*) was present on cells within the TNs (Figure S1G in Supplementary Material, *arrows*), and to a lesser extent, in cells within the peritumoral stroma (Figure S1G in Supplementary Material, brown, *arrowheads*).

Normal positive staining patterns for OCT4 (Figure S2A in Supplementary Material, brown), NANOG (Figure S2B in Supplementary Material, purple), SOX2 (Figure S2C in Supplementary Material, brown), c-Myc (Figure S2D in Supplementary Material, brown), and KLF4 (Figure S2E in Supplementary Material, purple) were demonstrated on human seminoma, normal skin, breast cancer, and prostate tissues, respectively. The negative control was a CAML sample DAB IHC stained using an IgG isotype (Figure S2F in Supplementary Material).

### IF IHC Staining

**Figure 1:**
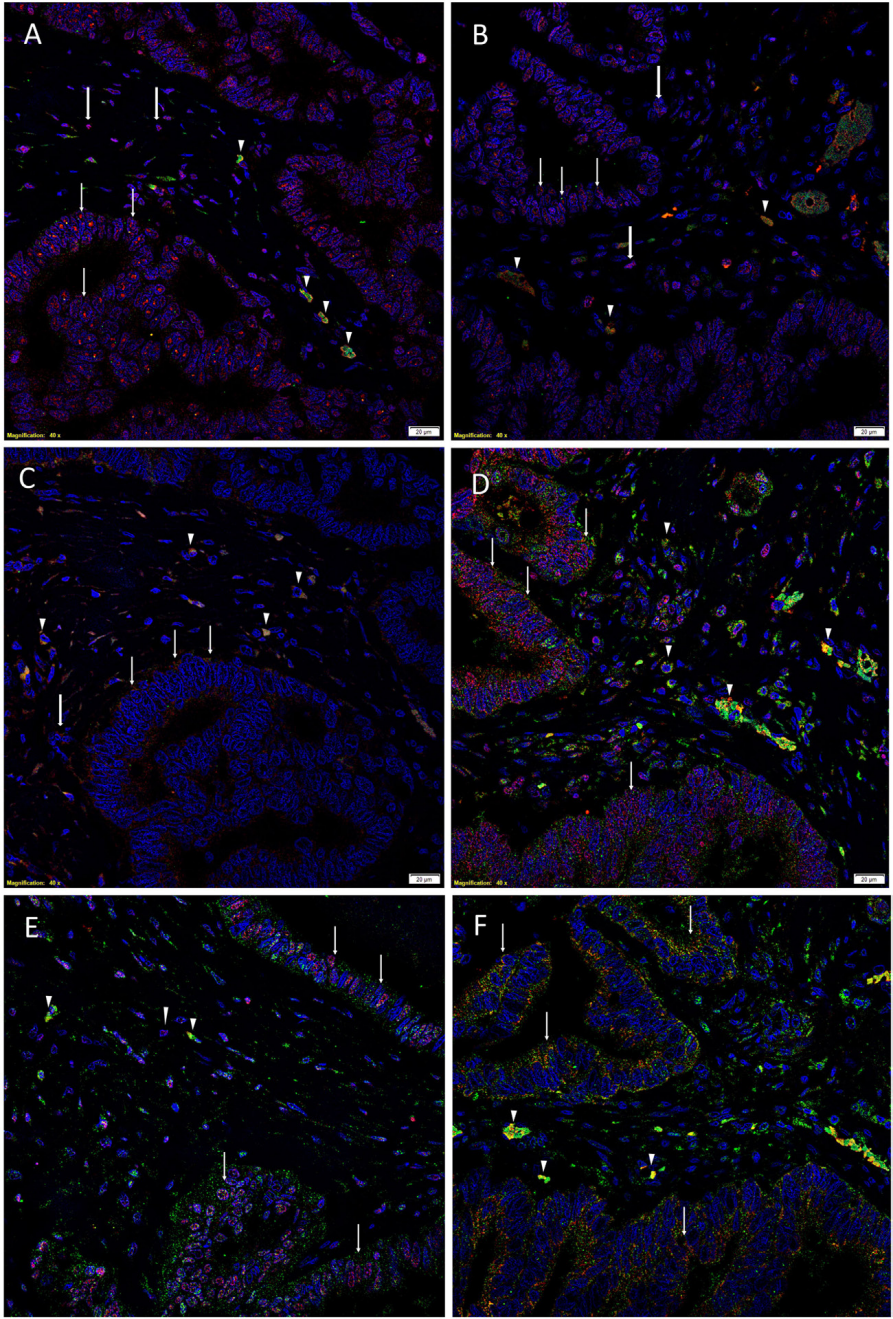
Representative immunofluorescence immunohistochemical-stained sections of colon adenocarcinoma metastasis to the liver showing the expression of SOX2 [**(A,D)**, red], NANOG [**(B,E)**, red], KLF4 [**(C,F)**, red], and c-Myc [**(D–F)**, green] on the cells within the tumor nests (TNs) [**(A–F)**, *thin arrows*] and those within the peritumoral stroma [**(A–F)**, *arrowheads*]. OCT4 [**(A–C)**, green] was expressed on the SOX2^+^ [**(A)**, green, *arrowheads*], NANOG^+^ [**(B)**, green, *arrowheads*], and KLF4^+^ [**(C)**, green, *arrowheads*] cells within the peritumoral stroma. There were also cells within the peritumoral stroma that expressed SOX2 [**(A)**, red, *thick arrows*], NANOG [**(B)**, red, *thick arrows*] and KLF4 [**(C)**, red, *thick arrows*] that did not express OCT4. c-Myc was expressed by cells within the TNs [**(D–F)**, green, *arrows*] and those within the peritumoral stroma [**(D–F)**, *arrowhead*s]. The c-Myc^+^ population within the peritumoral stroma also expressed SOX2 [**(D)**, red], NANOG [**(E)**, red], and KLF4 [**(F)**, red]. Cell nuclei were counterstained with 4′,6′-diamidino-2-phenylindole [**(A–F)**, blue]. Scale bars: 20 µm.

To demonstrate coexpression of the ESC markers, IF IHC staining was performed on two representative CAML samples from the original cohort of nine patients used for DAB IHC staining. SOX2 (Figures 1A,D, red), NANOG (Figures 1B,E, red), KLF4 (Figures 1C,F, red), and c-Myc (Figures 1D–F, green) were expressed on cells within the TNs (Figures 1A–F, *thin arrows*) and the peritumoral stroma (Figures 1A–F, *arrowheads*). OCT4 (Figures 1A–C, green) was expressed on the SOX2^+^ (Figure 1A, green, *arrowhead*s), NANOG^+^ (Figure 1B, green, *arrowheads*), and KLF4^+^ (Figure 1C, green, *arrowheads*) cells within the peritumoral stroma. Furthermore, there were cells within the peritumoral stroma that expressed SOX2 (Figure 1A, red, *thick arrows*), NANOG (Figure 1B, red, *thick arrows*), and KLF4 (Figure 1C, red, *thick arrows*) that did not express OCT4.

To determine the expression of c-Myc (Figures 1D–F, green), we performed dual staining with SOX2 (Figure 1D, red), NANOG (Figure 1E, red), and KLF4 (Figure 1F, red). c-Myc was expressed by cells within the TNs (Figures 1D–F, green, *arrows*) and cells within the peritumoral stroma (Figures 1D–F, *arrowhead*s). Intriguingly the c-Myc^+^ population within the peritumoral stroma also expressed SOX2 (Figure 1D, red), NANOG (Figure 1E, red), and KLF4 (Figure 1F, red).

Split images of all the stains presented in Figure 1 are shown in Figure S3 in Supplementary Material. Minimal staining was present on the negative control (Figure S3M in Supplementary Material), confirming the specificity of the primary antibodies used.

### NanoString mRNA Expression Analysis

**Figure 2:**
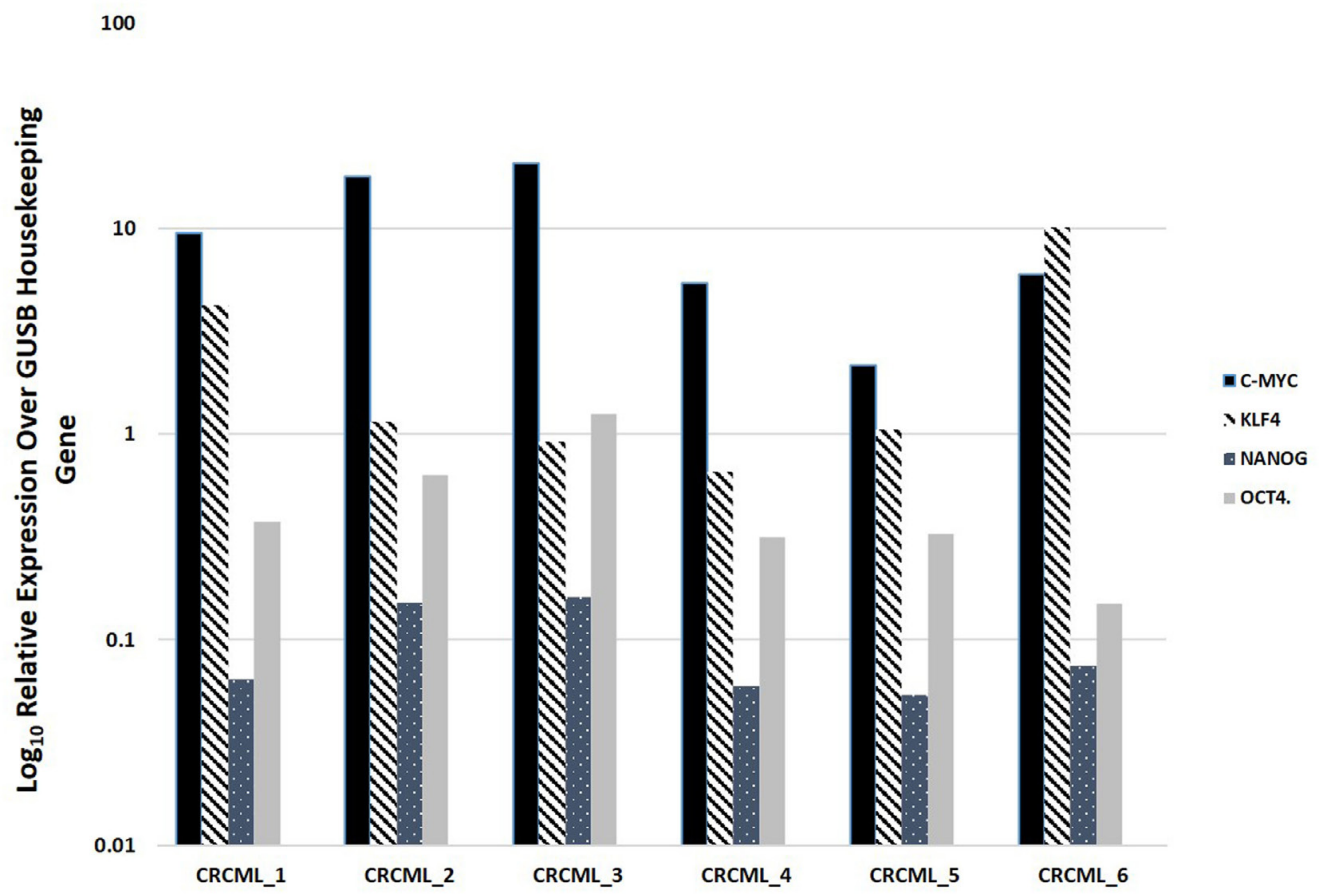
Expression of mRNA transcripts of c-Myc, KLF4, OCT4, and NANOG in colon adenocarcinoma metastasis to the liver samples from six patients, normalized against the GUSB housekeeping gene. The expression of SOX2 was below detectable level.

NanoString mRNA analysis confirmed expression of mRNA transcripts for OCT4, NANOG, KLF4, and c-Myc in all six CAML samples relative to the housekeeping gene GUSB (Figure 2). SOX2 mRNA transcript expression was below detectable levels in all CAML samples. RNA integrity analysis confirmed that two of the four samples had RIN > 7.

### Colormetric *In Situ* Hybridization

Colorimetric *in situ* hybridization demonstrated the presence of mRNA transcripts for OCT4 (Figure S4A in Supplementary Material, pink, *arrows*), SOX2 (Figure S4B in Supplementary Material, pink, *arrows*), NANOG (Figure S4C in Supplementary Material, pink, *arrows*), KLF (Figure S4D in Supplementary Material, pink, *arrows*), and c-Myc (Figure S4E in Supplementary Material, pink, *arrows*) in all six samples of CAML. Positive controls were human seminoma for OCT4 (Figure S5A in Supplementary Material, pink, *arrows*), normal skin for SOX2 (Figure S5B in Supplementary Material, pink, *arrows*), seminoma for NANOG (Figure S5C in Supplementary Material, pink, *arrows*), breast cancer for KLF4 (Figure S5D in Supplementary Material, pink, *arrows*), and normal prostate for c-Myc (Figure S5E in Supplementary Material, pink, *arrows*). The negative control was a sample of *Bacillus* (NM_L38424) (Figure S5F in Supplementary Material).

### Cell Counting and Statistical Analyses

**Figure 3:**
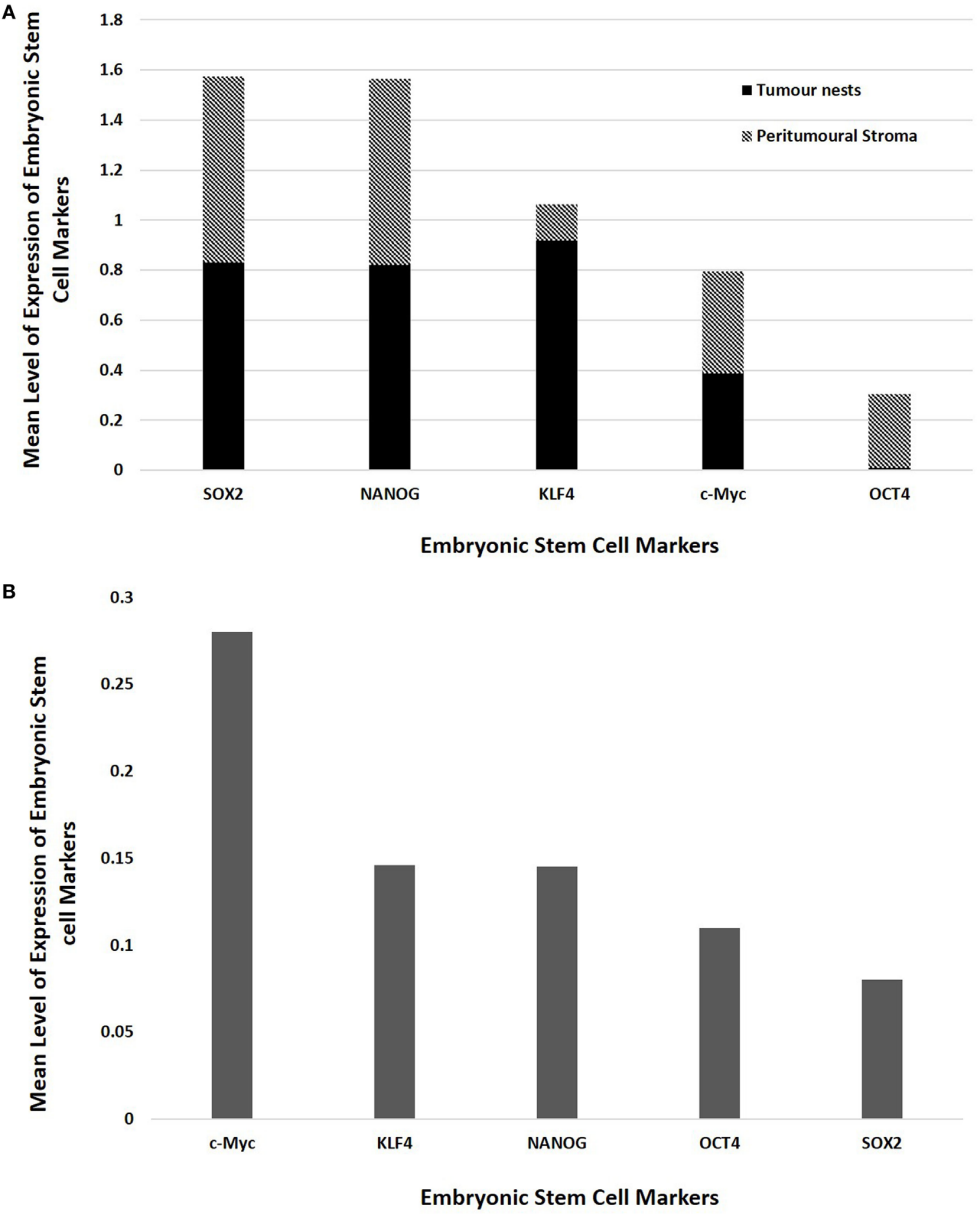
Bar graphs comparing mean positive expression of cells between the tumor nest and the peritumoral stromal subpopulations within colon adenocarcinoma metastasis to the liver for each embryonic stem cell (ESC) marker on 3,3-diaminobenzidine immunohistochemical staining **(A)** and comparing cumulative positive expression of cells in both the tumor nests and the peritumoral stroma for each ESC marker for colorimetric *in situ* hybridization **(B)**.

Statistical analyses of the results of cell counting of DAB-stained slides showed that there was statistically significant difference between the mean proportions of OCT4^+^, SOX2^+^, NANOG^+^, and KLF4^+^ cells in the peritumoral stroma compared to those within the TNs (*p* < 0.05), but there were no significant differences for c-Myc (*p* > 0.05) (Figure 3A). When comparing the total proportional of positively stained cells within the TNs and the peritumoral stroma for each marker, χ^2^ statistical analysis demonstrated a hierarchy of expression of these markers with increasing abundance: NANOG > SOX2 > KLF4 > c-Myc > OCT4. All differences were statistically significant (*p* < 0.01).

The means for the cell counts on CISH-stained slides (Figure 3B) analyzed by χ^2^ statistical analysis demonstrated the following hierarchy: c-Myc > KLF4 > NANOG > OCT4 > SOX2. All comparisons were highly statistically significant between markers (*p* < 0.0001) except for the comparison between KLF4 and NANOG which was not statistically significant (*p* = 0.884).

## Discussion

Colorectal cancer CSCs have been reported to express CD44 (28, 29). In this report, we observed its expression by cells within the TNs in CALM. Furthermore, this study demonstrates the novel finding of three putative subpopulations of CSCs within CAML: a SOX2^+^/NANOG^+^/KLF4^+^/c-Myc^+^/OCT4^+^ subpopulation, and a SOX2^+^/NANOG^+^/KLF4^+^/c-Myc^+^/OCT4^−^ subpopulation within the peritumoral stroma, and a SOX2^+^/NANOG^+^/KLF4^+^/c-Myc^+^/OCT4^−^ subpopulation within the TNs.

Interestingly the presence of SOX2 was demonstrated by IHC staining and CISH, but was not detectable by NanoString mRNA analysis. This discrepancy may be due to an observed 50% degradation of the mRNA during the process of NanoString mRNA analysis.

The relative abundance of the CSC markers used in this study may reflect the presence of a hierarchy of CSCs within this tumor.

**Figure 4:**
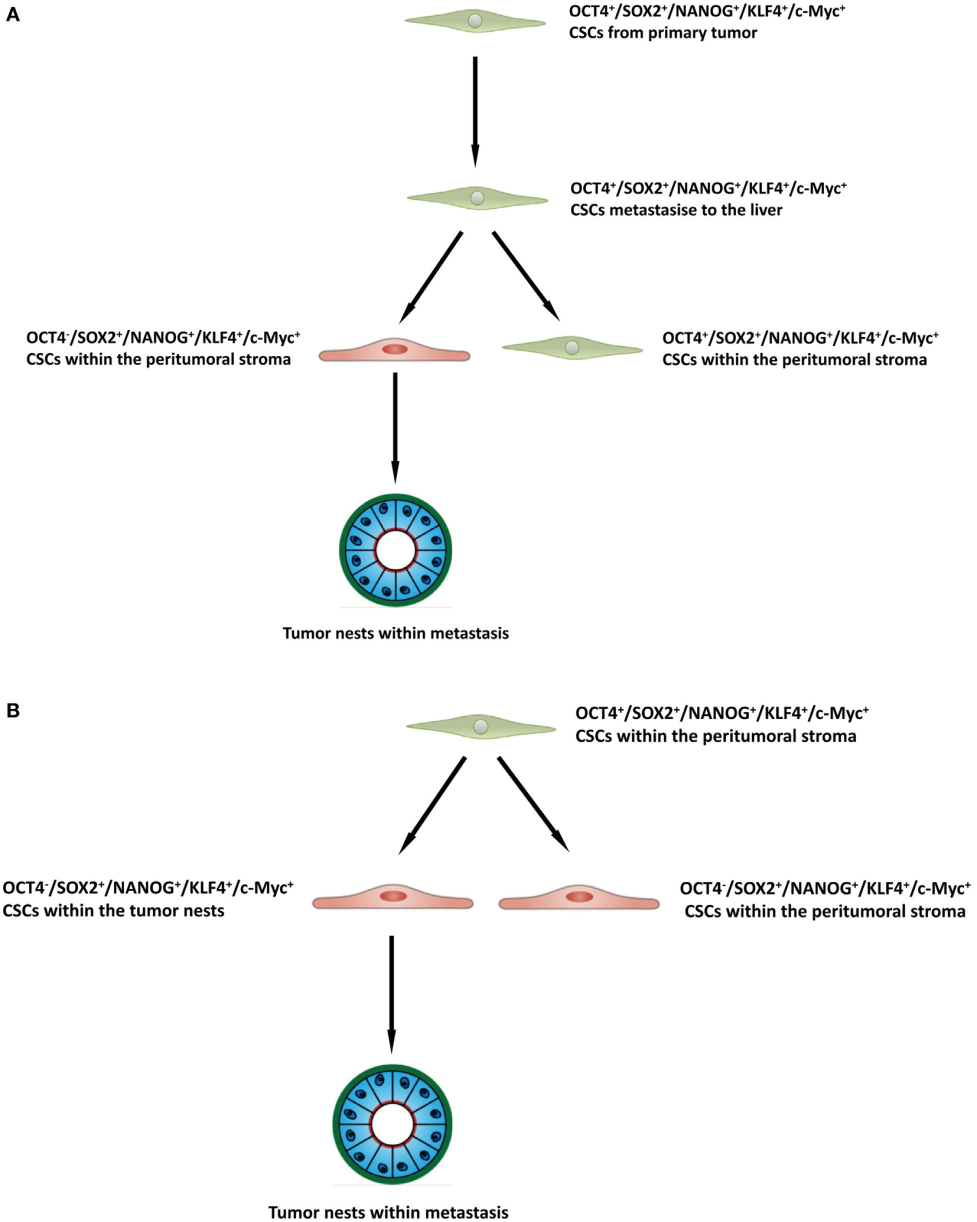
A schema proposing that cancer stem cells within the primary colorectal cancer undergo epithelial-mesenchymal transition and metastasize to the liver by seeding new tumors (metastases). These primitive cells can be identified by their expression of OCT4 which are present within the peritumoral stroma **(A)**, or the lack of expression of OCT4 on the cancer stem cells within the tumor nests **(B)**, and the downstream acquisition/loss of genes that results once the tumor has been seeded in the liver giving rise to the subpopulations seen in colorectal cancer metastasis to the liver.

The presence of three putative CSC subpopulations is intriguing. OCT was expressed only by cells within the peritumoral stroma, but not those within the TNs. We speculate these OCT4^+^ cells within the peritumoral stroma may represent the most primitive CSC subpopulation within CAML. This OCT4^+^ CSC subpopulation may give rise to the downstream OCT4^−^ subpopulations within the peritumoral stroma and those within the TNs (Figure 4A), although this putative presence of a CSC hierarchy remains the topic of further investigation.

Recent literature has reported on the heterogeneity of CSC (30, 31) and suggests a putative CSC hierarchy and the upregulation of OCT4 as potentially identifying the most primitive phenotype in the CSC population (32, 33).

It would be of great interest to carry out analysis of tissue samples of the primary CRCs that gave rise the liver metastases that we have studied, to determine the CSC phenotype. Unfortunately, this was not available from the cohort of patients included in this study, but will a subject of future investigation.

Epithelial–mesenchymal transition (EMT) is a pathway in which epithelial tumor cells deregulate the cytoskeletal structure to acquire mesenchymal morphology, with pathological implications in cancer metastasis and tumor invasion of the surrounding tissues (34, 35). The WNT/β catenin signaling pathway is activated in CRC, with growing evidence that this pathway interacts with the transcription and expression of embryonic transcription factors to modulate EMT (34, 35). The mesenchymal-epithelial transition (MET) pathway facilitates metastasis of tumor cells, which once integrated into a host tissue, their mesenchymal morphology transformed into epithelial phenotype (34, 35). Again, WNT/β catenin pathway is implicated in this morphological change, as inhibition of this pathway blocks MET (3, 4, 35).

It is exciting to speculate the presence of an OCT4^+^ CSC subpopulation within a primary CRC that undergoes an EMT process (36). The EMT process proposes that the epithelial CRC CSC acquire a mesenchymal phenotype which enables it to detach and metastasize to the liver *via* the portal circulation, in a similar way that circulating cancer cells are proposed to originate (37, 38). We speculate that these OCT4^+^ CSCs, upon “lodgement” in the liver then subsequently undergo MET (39), OCT4^−^ CSC subpopulation within the peritumoral stroma to complete the metastatic process. This latter CSC subpopulation then potentially gives rise to the OCT4^−^ CSCs within the TNs (Figure 4B). Alternatively, the “parent” metastatic OCT4^+^ CSCs may be seeded into the liver to form the TNs, which then gives rise to a gradual succession of primitive progenies (Figure 4B) with the OCT4^+^ being the ultimate end-product of this process. However, it is equally likely that the OCT4^+^ cells are not derived from the CRCLM, but instead are from the surrounding tissues and this clearly requires further investigation.

Recent studies have demonstrated a role for granulocyte macrophage colony-stimulating factor in inducing EMT in CRC, primarily being secreted by the colon epithelial cells (40, 41). Based on this study, it is exciting to speculate that the OCT4^+^ cells are a result of the process of EMT, although this is the topic of further investigation.

The finding of three putative subpopulations of CSC within CALM provides novel insight and warrants further investigation toward a better understanding of the biology of this common cancer.

## Ethics Statement

This study was carried out with the approval of the Central Health and Disability Ethics Committee (ref. no. 13/CEN/106) with written informed consent from all subjects in accordance with the Declaration of Helsinki.

## Author Contributions

TI and ST formulated the study hypothesis. TI, SW, and ST designed the study. HH, SW, HB, ST, and TI interpreted the DAB and IF IHC and the CISH data. HH, SW, ST, and TI interpreted the NanoString mRNA expression data. HH and SM performed cell counting on DAB IHC and CISH stained slides. RM conducted statistical analysis and interpreted the results. HH, ST, and TI drafted the manuscript. All authors commented on and approved the manuscript.

## Conflict of Interest Statement

The authors declare that the research was conducted in the absence of any commercial or financial relationships that could be construed as a potential conflict of interest. ST and TI are inventors of the PCT patent application for Cancer Diagnosis and Therapy (no. PCT/NZ2015/050108), and the PCT patent application for Cancer Therapeutic (US62/452479).

## Supplementary Material

Figure S1 A representative hematoxylin and eosin section of colon adenocarcinoma (CAML) metastasis to the liver showing tumor nests (TNs) in a glandular arrangement surrounded by the peritumoral stroma **(A)**. Representative 3,3-diaminobenzidine immunohistochemical-stained sections of CAML demonstrating membranous staining of CD44 [**(B)**, brown], and cytoplasmic staining of OCT4 [**(C)**, brown] in the peritumoral stroma. Nuclear expression of NANOG on the cells within the TNs [**(D)**, brown, *arrows*] and those within the peritumoral stroma [**(D)**, brown, *arrowheads*]. SOX2 was expressed on the nuclei of the cells within the TNs [**(E)**, brown, *arrows*] and those within the peritumoral stroma [**(E)**, brown, *arrowheads*]. C-Myc was expressed in the nuclei of cells within the TNs [**(F)**, brown, *arrows*] and those within the peritumoral stroma [**(F)**, brown, *arrowheads*]. KLF4 was expressed in the cytoplasm of the cells within the TNs [**(G)**, brown, *arrows*] and, to a lesser extent, those within the peritumoral stroma [**(G)**, brown, *arrowheads*]. Nuclei were counter-stained with hematoxylin [**(A–G)**, blue]. Original magnification: 400×.

Figure S2 3,3-Diaminobenzidine immunohistochemical-stained sections of human seminoma for OCT4 [**(A)**, brown] and NANOG [**(B)**, brown], skin for SOX2 [**(C)**, brown], normal prostate for c-Myc [**(D)**, Brown] and breast cancer for KLF4 [**(E)**, brown] and staining with an IgG isotype antibody provided an appropriate negative control **(F)**. Nuclei were counter-stained with hematoxylin [**(A–F)**, blue]. Original magnification: 400×.

Figure S3 Split images of immunofluorescent immunohistochemical-stained sections of colon adenocarcinoma metastasis to the liver (CAML) shown in Figure 2 demonstrating coexpression of OCT4 [**(A,C,E)**, green] with SOX2 [**(B)**, red], NANOG [**(D)**, red] and KLF4 [**(F)**, red]; c-Myc [**(G,I,K)**, green] and SOX2, [**(H)**, red], NANOG [**(J)**, red] and KLF [**(L)**, red]. A negative control **(M)** to test the specificity of the fluorescent secondary antibodies is performed on a section of CRCML. Cell nuclei were counter-stained with 4′,6-diamidino-2-phenylindole [**(A–M)**, blue]. Scale bars: 20 µm.

Figure S4 representative colorimetric *in situ* hybridization stained sections of colon adenocarcinoma metastasis to the liver demonstrating mRNA expression of OCT 4 [**(A)**, pink], SOX2 [**(B)**, pink], NANOG [**(C)**, pink], KLF4 [**(D)**, pink], and c-Myc [**(E)**, pink]. Nuclei were counter-stained with hematoxylin [**(A–E)**, blue]. Original magnification: 1,000×.

Figure S5 Positive human control colorimetric *in situ* hybridization stained sections of seminoma for NANOG [**(A)**, pink], skin for SOX2 [**(B)**, pink], seminoma for OCT4 [**(C)**, pink], normal prostate for c-Myc [**(D)**, pink], and breast cancer [**(E)**, pink]. Negative control **(F)** performed on a section of *Bacillus* confirmed the specificity of the secondary antibody. Nuclei were counter-stained with hematoxylin [**(A–F)**, blue]. Original magnification: 1,000×.
