# Supplementary figures and images for: Characterization of Cancer Stem Cells in Colon Adenocarcinoma Metastasis to the Liver
Source: Front Surg. 2018 Jan 22;4:76. doi: 10.3389/fsurg.2017.00076 (PMC5786574; doi:10.3389/fsurg.2017.00076)

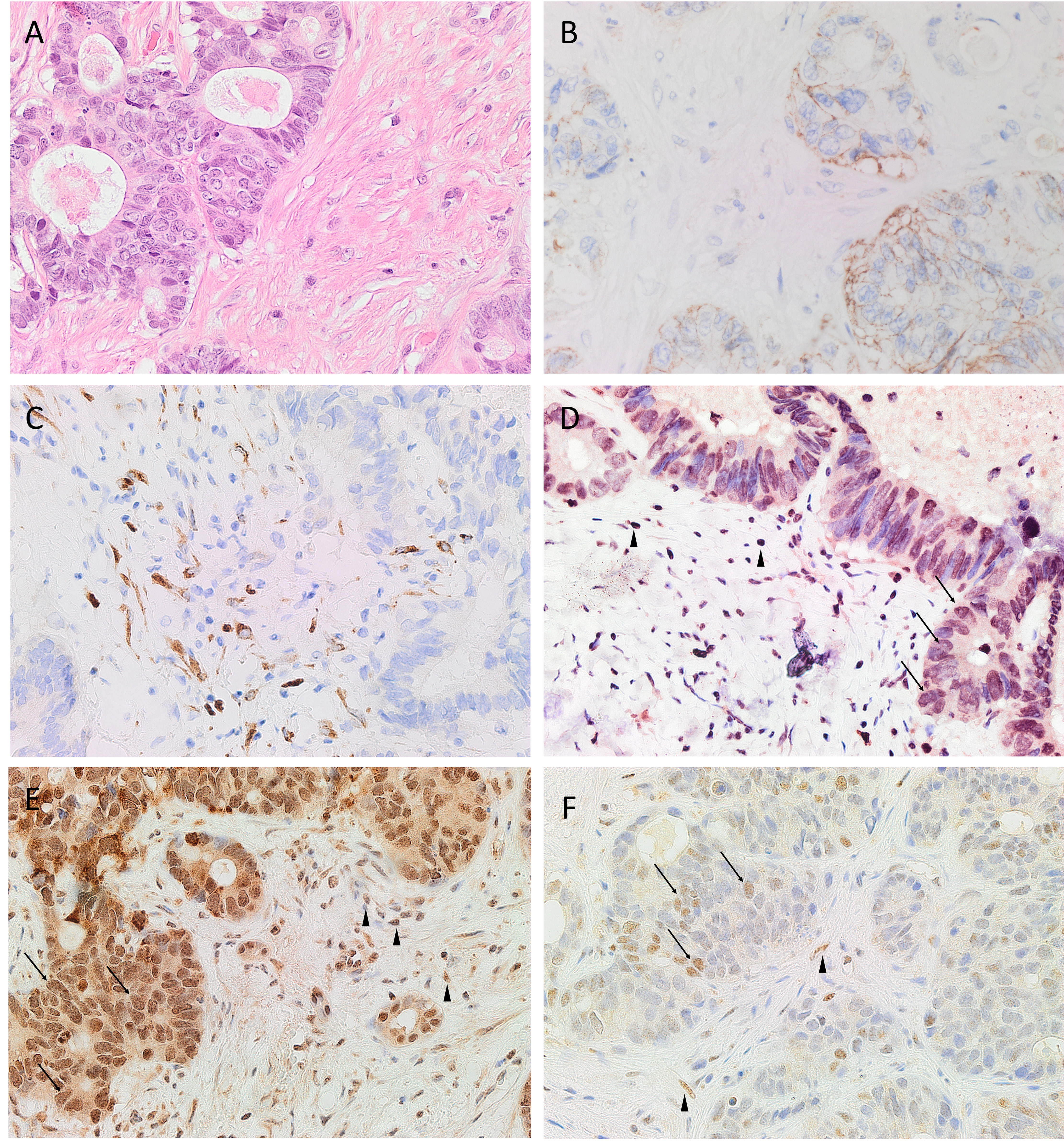

Supplement: Figure S1 — A representative hematoxylin and eosin section of colon adenocarcinoma (CAML) metastasis to the liver showing tumor nests (TNs) in a glandular arrangement surrounded by the peritumoral stroma (A). Representative 3,3-diaminobenzidine immunohistochemical-stained sections of CAML demonstrating membranous staining of CD44 [(B), brown], and cytoplasmic staining of OCT4 [(C), brown] in the peritumoral stroma. Nuclear expression of NANOG on the cells within the TNs [(D), brown, arrows] and those within the peritumoral stroma [(D), brown, arrowheads]. SOX2 was expressed on the nuclei of the cells within the TNs [(E), brown, arrows] and those within the peritumoral stroma [(E), brown, arrowheads]. C-Myc was expressed in the nuclei of cells within the TNs [(F), brown, arrows] and those within the peritumoral stroma [(F), brown, arrowheads]. KLF4 was expressed in the cytoplasm of the cells within the TNs [(G), brown, arrows] and, to a lesser extent, those within the peritumoral stroma [(G), brown, arrowheads]. Nuclei were counter-stained with hematoxylin [(A–G), blue]. Original magnification: 400×. [file Image_1.JPEG]

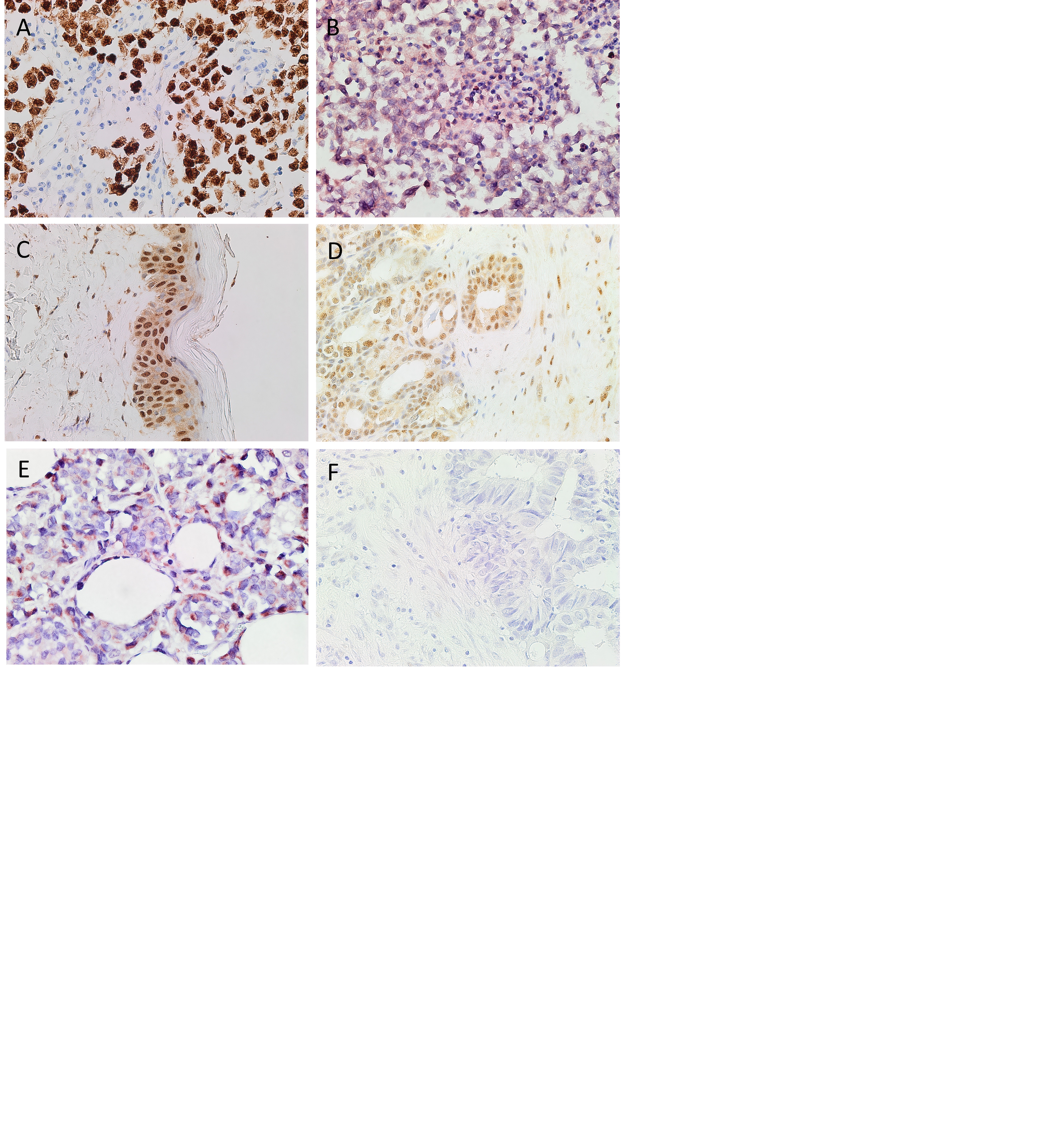

Supplement: Figure S2 — 3,3-Diaminobenzidine immunohistochemical-stained sections of human seminoma for OCT4 [(A), brown] and NANOG [(B), brown], skin for SOX2 [(C), brown], normal prostate for c-Myc [(D), Brown] and breast cancer for KLF4 [(E), brown] and staining with an IgG isotype antibody provided an appropriate negative control (F). Nuclei were counter-stained with hematoxylin [(A–F), blue]. Original magnification: 400×. [file Image_2.JPEG]

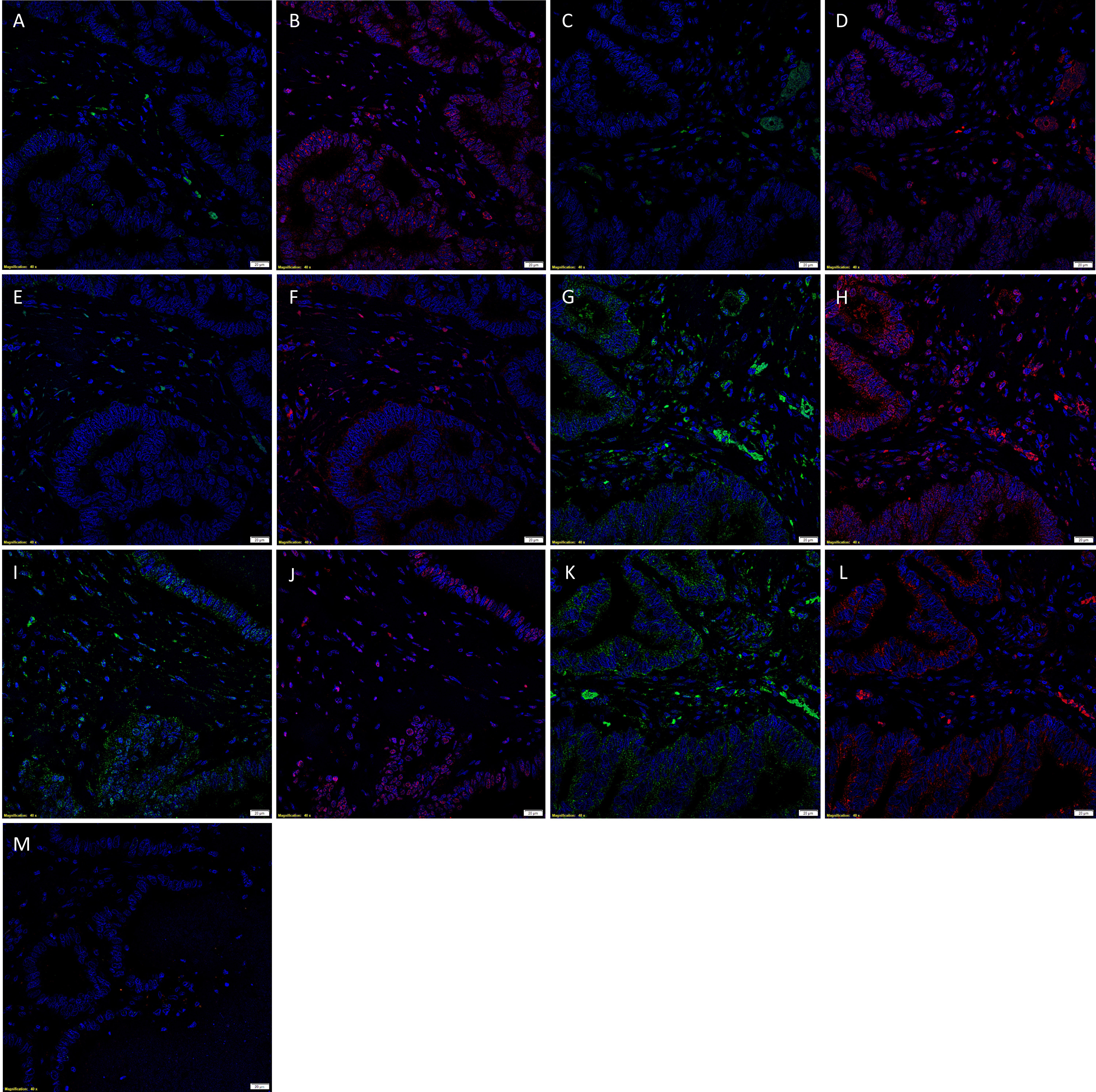

Supplement: Figure S3 — Split images of immunofluorescent immunohistochemical-stained sections of colon adenocarcinoma metastasis to the liver (CAML) shown in Figure 2 demonstrating coexpression of OCT4 [(A,C,E), green] with SOX2 [(B), red], NANOG [(D), red] and KLF4 [(F), red]; c-Myc [(G,I,K), green] and SOX2, [(H), red], NANOG [(J), red] and KLF [(L), red]. A negative control (M) to test the specificity of the fluorescent secondary antibodies is performed on a section of CRCML. Cell nuclei were counter-stained with 4′,6-diamidino-2-phenylindole [(A–M), blue]. Scale bars: 20 µm. [file Image_3.JPEG]

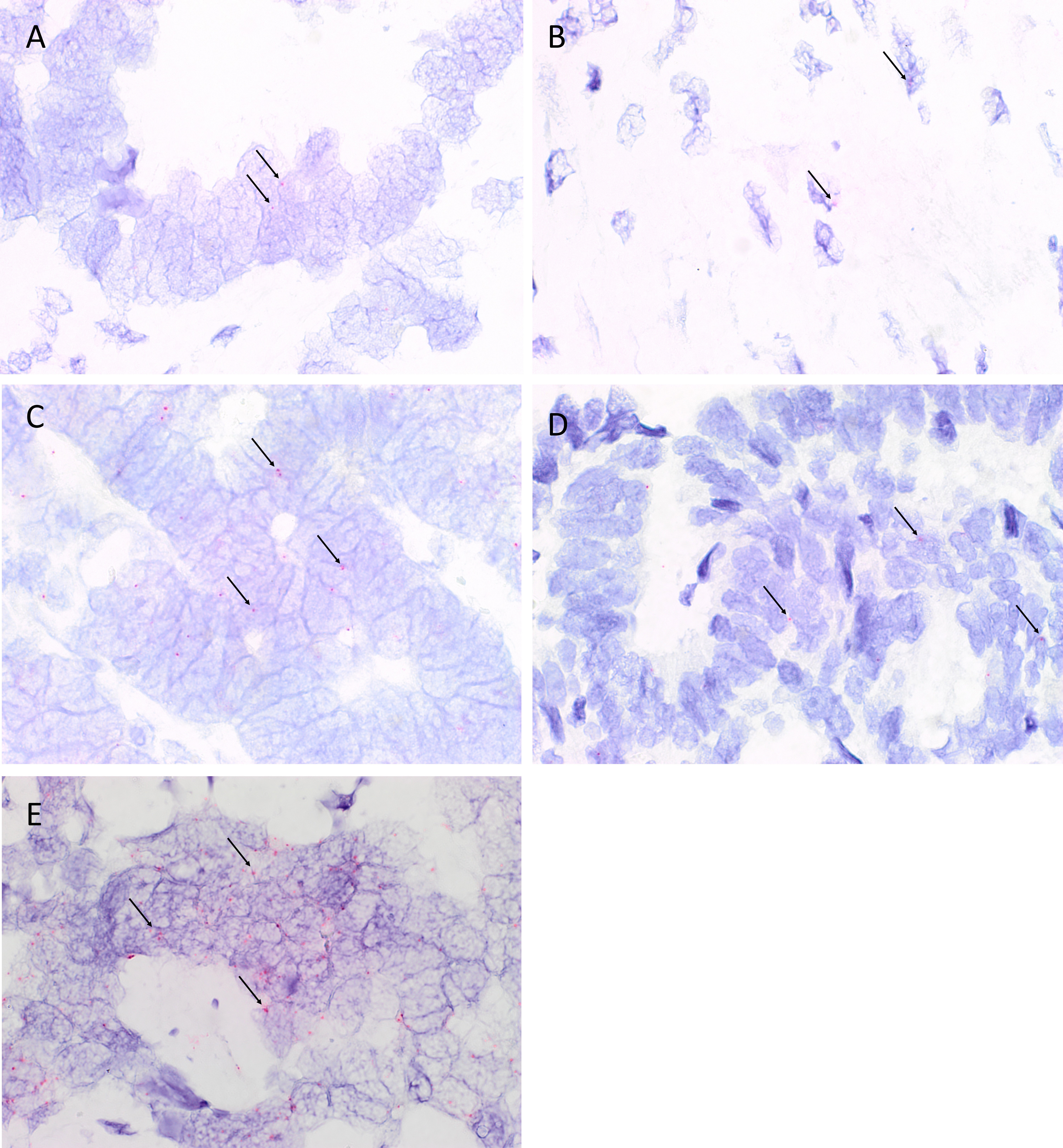

Supplement: Figure S4 — representative colorimetric in situ hybridization stained sections of colon adenocarcinoma metastasis to the liver demonstrating mRNA expression of OCT 4 [(A), pink], SOX2 [(B), pink], NANOG [(C), pink], KLF4 [(D), pink], and c-Myc [(E), pink]. Nuclei were counter-stained with hematoxylin [(A–E), blue]. Original magnification: 1,000×. [file Image_4.JPEG]

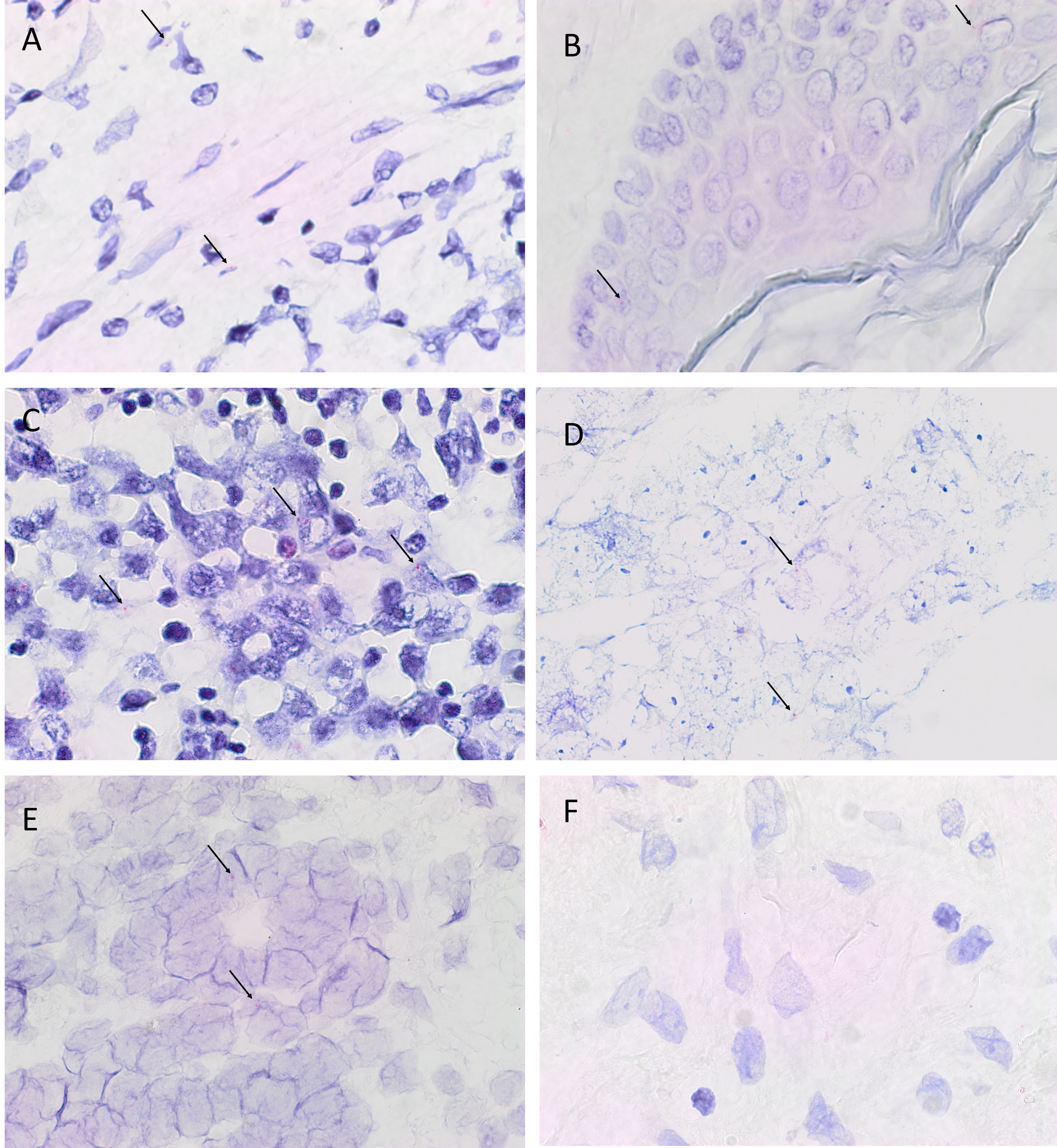

Supplement: Figure S5 — Positive human control colorimetric in situ hybridization stained sections of seminoma for NANOG [(A), pink], skin for SOX2 [(B), pink], seminoma for OCT4 [(C), pink], normal prostate for c-Myc [(D), pink], and breast cancer [(E), pink]. Negative control (F) performed on a section of Bacillus confirmed the specificity of the secondary antibody. Nuclei were counter-stained with hematoxylin [(A–F), blue]. Original magnification: 1,000×. [file Image_5.JPEG]
